# Disenfranchised grief and early pregnancy loss – apropos a clinical case

**DOI:** 10.1192/j.eurpsy.2021.2202

**Published:** 2021-08-13

**Authors:** S. Freitas Ramos, B. Jesus, M.I. Fonseca Marinho Vaz Soares, J. Martins Correia, J. Mendes

**Affiliations:** Department Of Psychiatry And Mental Health, Local Health Unit of Guarda, Guarda, Portugal

**Keywords:** Grief, early pregnancy loss

## Abstract

**Introduction:**

Perinatal death includes losses such as ectopic pregnancies, miscarriages, stillbirths and neonatal deaths. Perinatal loss has well documented negative effects on the health of the bereaved parents. Early pregnancy loss (EPL) is the spontaneous death of a fetus within the first 20 weeks of gestation.

**Objectives:**

To describe a clinical case of disenfranchised grief following EPL and to review the literature.

**Methods:**

We reviewed the clinical file of a patient presenting to the psychiatry outpatient clinic with disenfranchised grief. We conducted a non-systematic review on PubMed and Google Scholar.

**Results:**

A 29-years-old female patient presents to the outpatient clinic with depressive symptoms and thoughts of death. The symptoms had begun 4 months earlier, following the loss of pregnancy at 14 weeks. She felt her grief was not accepted by her family and social network. Progressively, her relationships deteriorated, and she felt more and more isolated. She experienced marked difficulty in caring for her older child. Compared to other types of mourning, the loss of a child is associated with grief experience that is particularly severe and complicated. Despite the high prevalence of EPL, many women suffer in silence due to the common belief these losses are insignificant and may develop complicated grief.

**Conclusions:**

Perinatal loss of an infant has the potential to have a large impact on the mental health of the bereaved parents. Literature on the efficacy of different interventions is still scarce. Further studies are necessary on prevention strategies and interventions for parents already suffering from complicated grief or depressive disorders.

**Disclosure:**

No significant relationships.

